# Comparison of Bond Strength of Different Eighth-Generation Adhesives in Orthodontic Bonding

**DOI:** 10.7759/cureus.86439

**Published:** 2025-06-20

**Authors:** Meghali Garg, Mandeep K Bhullar, Gaurav Ahuja, Abida Parveen, Naman Mittal, Stela Kapoor

**Affiliations:** 1 Department of Orthodontics and Dentofacial Orthopedics, Maharishi Markandeshwar College of Dental Sciences and Research, Ambala, IND

**Keywords:** adhesive remnant index, dentistry, eight-generation bonding agent, orthodontic bonding, self-etch adhesives, shear bond strength

## Abstract

Objective: This in vitro study aims to compare the shear bond strength (SBS) and Adhesive Remnant Index (ARI) values of various eighth-generation adhesives used in orthodontic bonding.

Materials and methodology: A total of 40 extracted human premolars were randomly divided into four groups; (i) Group 1: 3M Unitek Transbond-XT (fifth-generation adhesive, conventional etch, 3M Electro & Communication India Private Limited, Bengaluru, India) (ii) Group 2: 3M ESPE Single Bond Universal (eighth-generation, 3M Electro & Communication India Private Limited, Bengaluru, India), (iii) Group 3: Tetric-N-Bond Universal (eighth-generation, Ivoclar Vivadent, Schaan, Liechtenstein), and (iv) Group 4: G-Premio Bond (eighth-generation, GC Corporation, London, UK). The premolars, stored in saline water, were bonded with stainless steel brackets using respective bonding agents and 3M Unitek Transbond-XT composite. The SBS values were tested using an Instron universal testing machine (Asian Test Equipments, Ghaziabad, India). Post-debonding, ARI scores for each group were evaluated using a stereomicroscope at 10X magnification.

Results: The SBS values for the adhesives were 3M ESPE Single Bond Universal (2.29 ± 1.12 MPa), Tetric-N-Bond Universal (5.26 ± 2.6 MPa), G-Premio Bond (5.07 ± 1.72 MPa), and 3M Unitek Transbond-XT (10.22 ± 4.83 MPa). ARI scores differed across groups, with most samples showing a score of 2 and only the 3M Unitek Transbond-XT group recording an ARI score of 3. The Chi-square value was 19.42 (p = 0.02), indicating a moderately significant difference.

Conclusion: This study concluded that while eighth-generation adhesives like Tetric-N-Bond Universal and G-Premio Bond offer advantages in enamel protection and cleanup due to lower ARI scores, their SBS was lower than that of 3M Unitek Transbond-XT, which performed significantly better.

## Introduction

Successful orthodontic treatment depends on the accurate application of continuous forces using orthodontic appliances, with bracket bonding achieved through micromechanical adhesion using resin-based adhesives that penetrate the etched enamel surface. An ideal adhesive should infiltrate enamel porosities effectively, be easy to handle, and remain dimensionally stable to ensure long-term success [1]. Over the years, advancements in adhesive technology have significantly transformed dentistry, particularly in cosmetic procedures. Adhesive systems have evolved from being largely ineffective in the 1970s to the more reliable total-etch and self-etch systems used today. Early adhesives were considered ineffective primarily due to their high moisture sensitivity, which made them prone to failure when exposed to even minimal moisture during bonding. Additionally, they lacked sufficient bond strength to withstand the mechanical forces encountered during orthodontic treatment, often resulting in bracket failures or restoration dislodgment. These adhesives were also highly technique-sensitive, requiring meticulous moisture control and consistent application, which made achieving reliable results challenging. Furthermore, their chemical composition lacked the advanced monomers and hydrophilic components found in modern adhesives, limiting their ability to form a stable hybrid layer and maintain durable adhesion to both enamel and dentin [2]. Despite these improvements, challenges remain, particularly due to water absorption and the complex oral environment, which includes moisture, temperature fluctuations, food, chewing forces, and varying pH levels [3]. Universal adhesives represent the latest innovation, aiming to simplify and enhance bonding protocols, and may mark the next major development in adhesive dentistry [2].

In orthodontic bonding, the enamel is etched with 37% phosphoric acid to create a "honeycomb" pattern that increases surface area and enhances mechanical retention of adhesives, leading to stronger bracket attachment. Etching also improves enamel's ability to bond with water-based adhesives by removing its hydrophobic layer. However, over-etching or improper technique can cause enamel damage, such as loss, weakening, or fractures during debonding. Acid etching can be uncomfortable for patients, removes the fluoride-rich enamel layer, and increases the risk of demineralization, white spot lesions, and enamel decalcification [4]. To address these drawbacks, self-etch primers were introduced. These combine etching and priming into a single step, reducing chair time and minimizing procedural errors while also being less damaging to enamel [5].

Adhesive systems in orthodontics have evolved through eight generations, each improving bond strength and simplifying application. The fourth-generation system used separate etchant, primer, and adhesive, while the fifth-generation combined them into a single system, achieving the highest shear bond strength (SBS) (15.49 ± 2.55 MPa) [6,7,8]. The sixth-generation system involves a self-etch primer and has a mean SBS less than that of the fifth-generation of around 11.57 ± 1.99 MPa [7-9]. Further attempts were made to develop a product with improved SBS values. This led to the introduction of the seventh-generation bonding system, which showed SBS of 13.51±2.45 MPa [7,9]. Although these were superior to the sixth generation, they still fell short of the fifth-generation bonding system.

The eighth-generation systems, the latest advancement, offer one-step, self-etch bonding with enhanced durability and versatility. These systems include a mix of advanced functional monomers, 4-MTA (4-methacryloxyethyl trimellitic anhydride), 10-MDP (10-methacryloyloxydecyl dihydrogen phosphate), and 10-MDPT (10-methacryloyloxydecyl dihydrogen thiophosphate), omitting the HEMA (2-hydroxyethyl methacrylate) component to reduce degradation. The 10-MDP monomer allows strong, hydrolytically stable ionic bonds with hydroxyapatite, improving longevity [10]. Eighth-generation adhesives also incorporate nano-fillers (~12 nm) that enhance adhesive penetration, bond strength, and stress distribution. These fillers also enable dual-curing and improve shelf life. Despite their promise, more long-term research is needed, and correct handling remains crucial for optimal outcomes [8,11].

A key factor in orthodontic bonding is SBS, which measures resistance to chewing and functional forces. An SBS of 5.9-7.8 MPa (Reynolds) is considered sufficient, but higher values offer better retention, though overly strong bonds risk enamel damage during bracket removal [9,12]. After debonding, the leftover adhesive must be carefully cleaned to avoid enamel roughness, staining, and plaque buildup. The Adhesive Remnant Index (ARI), designed by Artun and Bergland [13], helps assess where the bond failure occurred, guiding safer and more effective enamel restoration post-treatment.

Various eighth-generation universal bonding agents have been developed to simplify and enhance orthodontic bonding. The 3M ESPE Single Bond Universal (3M Electro & Communication India Private Limited, Bengaluru, India) also known as the 3M Scotchbond Universal bonding agent globally is a versatile agent compatible with all etching techniques and a wide range of substrates, including enamel, dentin, glass ionomer, ceramics, and metals. It primarily supports light-cured materials but requires an activator for self- or dual-cure applications [14]

Tetric N-Bond Universal (Ivoclar Vivadent, Schaan, Liechtenstein) is another light-cured, single-component adhesive suitable for both direct and indirect restorations, offered in the efficient VivaPen format. It includes methacrylates, ethanol, and silicon dioxide. Methacrylates serve as the core resin monomers, facilitating strong micromechanical interlocking by penetrating the collagen network of the demineralized dentin, resulting in durable adhesion to both enamel and dentin. Ethanol acts as a solvent, reducing surface tension to ensure deeper penetration into etched dentin tubules, which improves hybrid layer formation and minimizes adhesive failure, especially in moist environments. Additionally, ethanol aids in displacing water from the dentin surface, enhancing moisture tolerance. Silicon dioxide functions as a filler that improves the viscosity and mechanical strength of the adhesive, contributing to increased wear resistance and a thin, uniform adhesive layer that does not interfere with indirect restoration placement [15].

G-Premio Bond (GC Corporation, London, UK) is another eighth-generation adhesive containing functional monomers (4-MTA, MDP, MDTP) that ensure strong bonds with tooth structure and indirect materials like zirconia and metals. It is compatible with all etch modes and aids in managing hypersensitivity and repairing ceramics [16]. These one-step adhesives reduce chairside time, an important advantage in orthodontics. However, limited data are available on their SBS in orthodontic use. Thus, the current study aims to compare the SBS and ARI of three eighth-generation adhesives, the G-Premio Bond, Tetric N-Bond Universal, and 3M ESPE Bond Universal, against a conventional fifth-generation adhesive, 3M Unitek Transbond-XT (3M Electro & Communication India Private Limited, Bengaluru, India), to evaluate their effectiveness in orthodontic bracket bonding.

## Materials and methods

**Figure 1:**
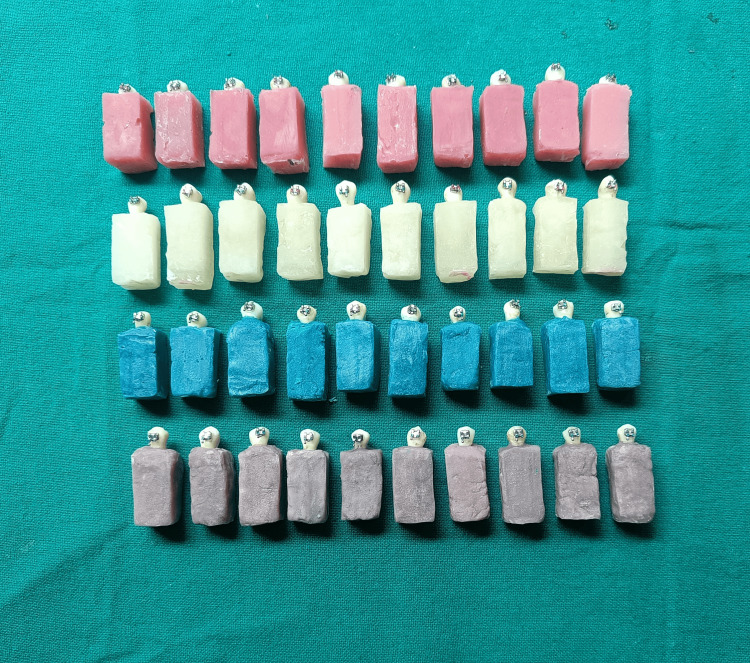
Bonded samples for testing Group-1 (Pink): 3M Unitek Transbond-XT Group-2 (Clear): 3M ESPE Single Bond Universal Group-3 (Aqua Green): Tetric-N-Bond Universal Group-4 (Grey): G-Premio Bond

The sample size calculation was done using OpenEpi, Version 3.01 (https://www.openepi.com) software to achieve a statistical power of 80% with a significance level of 5%, taking into account a 1.7 standard deviation. A sample of 10 premolar crowns per group was estimated. Samples were randomly divided into four subgroups (n=10) as follows: (i) Group 1: Ten premolars (color-coded by pink color acrylic blocks) were bonded with metal brackets using 3M Unitek Transbond-XT (fifth generation) bonding agent, (ii) Group 2: Ten premolars (color-coded by clear color acrylic blocks) were bonded with metal brackets using the 3M ESPE Single Bond Universal (eighth generation) bonding agent, (iii) Group 3: Ten premolars (color-coded by aqua green color acrylic blocks) were bonded with the Tetric-N-Bond Universal (eighth generation) bonding agent, (iv) Group 4: Ten premolars (color-coded by grey color acrylic blocks) were bonded with the G-Premio Bond (eighth-generation) bonding agent (Figure 1). Thus, the study consisted of 40 extracted human premolars.

Inclusion criteria

Morphologically sound premolars, premolars with intact surfaces, absence of any type of developmental defect, absence of dental caries or restoration.

Exclusion criteria

Fractured crown, any crack visible to the naked eye, dental caries, any restorations, developmental defects such as enamel hypoplasia, gross irregularities, fluorosed teeth.

Etching, conditioning, and bonding steps for tooth crown surfaces

Etching and Conditioning Using the Primer Agent

Group 1: Teeth in this group were treated as the control group and were etched with 37% orthophosphoric acid (Avue etch, BLV Healthcare, India) and rinsed. Then the conventional 3M Unitek Transbond-XT bonding agent was applied and light-cured for 10 seconds.

Group 2: Teeth in this group were treated with the self-etch mode of 3M ESPE Single Universal Bond. It was applied to the entire tooth and rubbed for 20 seconds, air-dried for five seconds, and then light-cured for 10 seconds.

Group 3: Teeth in this group were directly treated with the self-etch mode of the Tetric-N-Bond Universal bonding agent. A coat of adhesive was applied and agitated for 20 seconds, air-dried for five seconds, and then light-cured for 10 seconds.

Group 4: Teeth in this group were treated with the self-etch mode of the G-Premio Bond. The 35-second procedure was followed for application, i.e., the adhesive was rubbed for 20 seconds, the surface was air dried for five seconds, and then light cured for 10 seconds.

**Figure 2:**
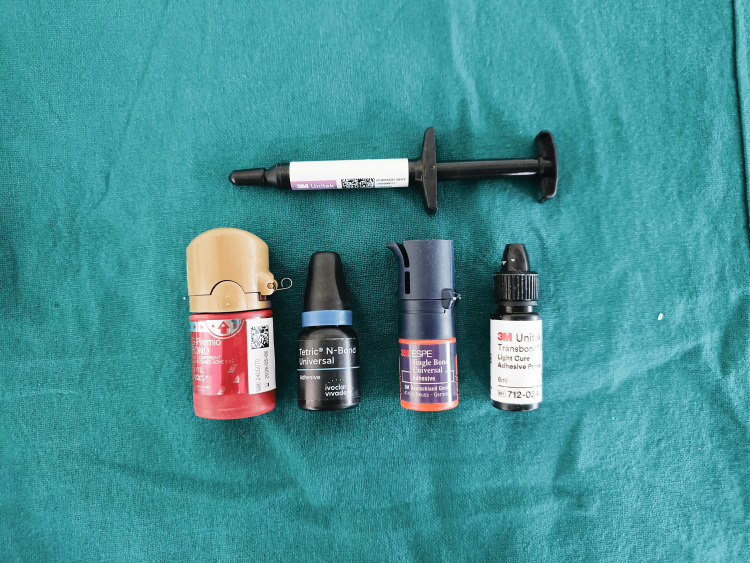
Bonding agents and composite adhesives used in this study

All the bonding agents and composite adhesives used are shown in Figure 2.

Bracket Bonding

After applying light-cure adhesive resin (3M Unitek Transbond-XT) over the base of each bracket, 0.022 MBT stainless steel maxillary premolar brackets (Ortho Organizer, Ortho Organizers Inc., Carlsbad, CA, USA) were bonded at the center of the buccal surface of crowns in all the groups (Figure 1).

Flash was removed from the crown surface around brackets, and each bracket was cured using a light curing unit (iLed, Guilin Woodpecker Medical Instrument Co., Ltd, Guilin, Guangxi Zhuang Autonomous Region, China, 1200-2000 mW/cm^2^) for 40 seconds per bracket (20 seconds mesially and 20 seconds distally). All the steps during bonding were strictly done according to the manufacturer’s guidelines for the particular material. The sample was again stored in saline water till the time of testing.

**Figure 3:**
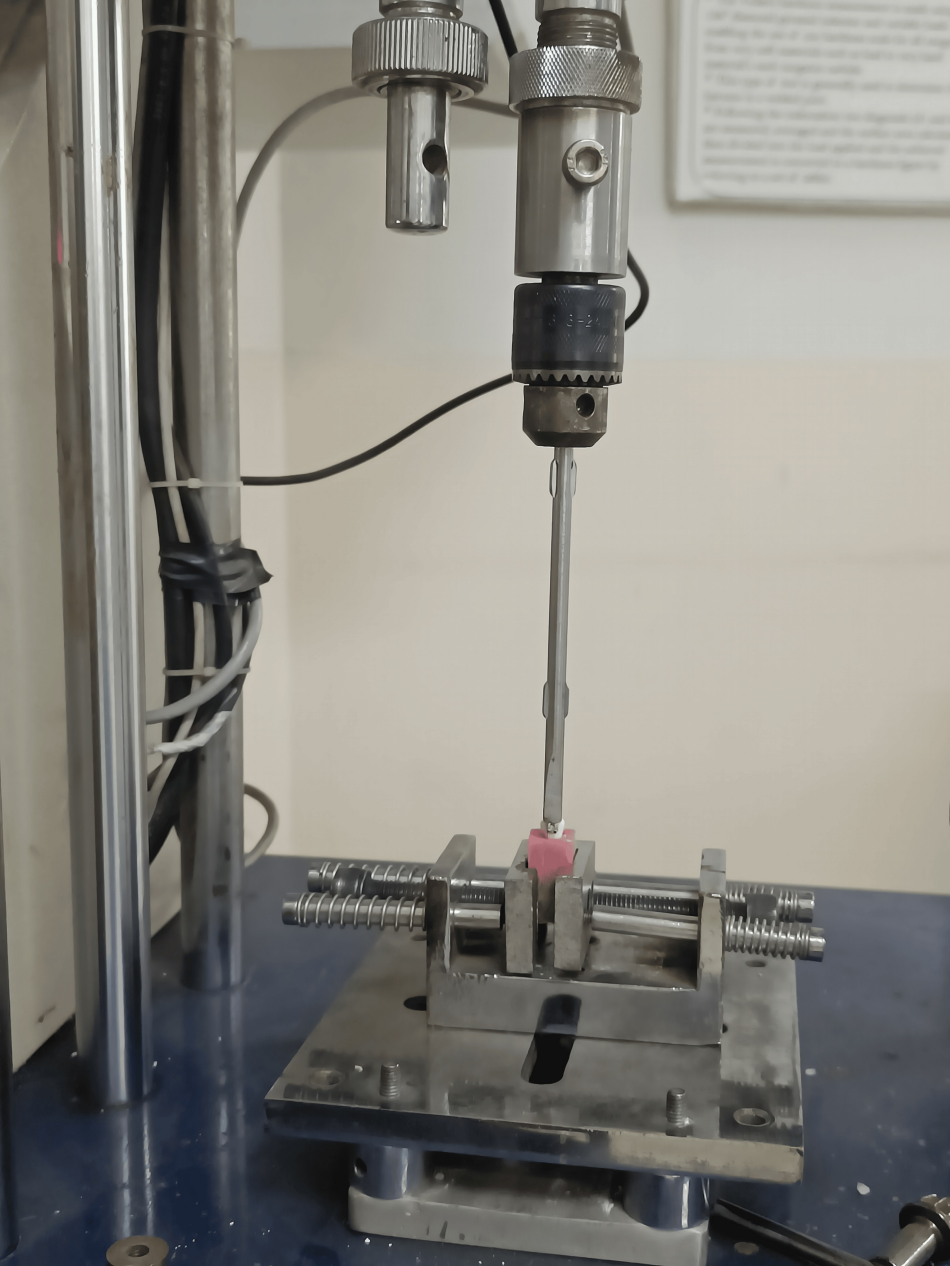
Debonding of samples done using universal testing machine

SBS of brackets bonded to the crown surface was determined with the help of a universal testing machine (Instron, Asian Test Equipments, Ghaziabad, India). The load was applied in the occluso-gingival direction at the bracket-crown interface with a cross-head speed adjusted to 1 mm/min to shear the bracket off the crown surface. The force at which the bracket debonded was recorded in Newton (N) on the monitor attached to the universal testing machine (Figure 3).

**Figure 4:**
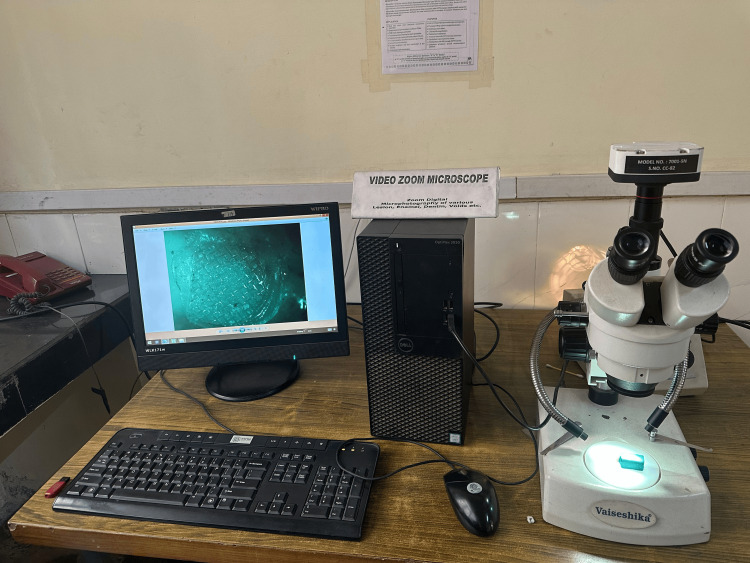
Samples being visualized for ARI index at 10X magnification using Videozoom stereomicroscope ARI: Adhesive Remnant Index

After debonding the brackets, all specimens were collected and visually examined under a Videozoom stereomicroscope (Videozoom stereomicroscope, Model No. 7001-5N, IS Capture Software, Vaiseshika Electron Devices, Ambala, Haryana, India) at 10X magnification to determine the amount of resin remaining on the tooth surface which helps to detect the site of bond failure (Figure 4).

The criteria for ARI scoring designed by Artun and Bergland [13] were used in this study. The criteria are as follows: (1) score 0 - no adhesive remains on the tooth surface; (2) score 1 - less than 50% of the adhesive remains on the tooth; (3) score 2 - more than 50% of the adhesive remains on the tooth; (3) score 3 - the entire adhesive remains on the tooth, showing a clear imprint of the bracket mesh.

Statistical analysis

All the data was compiled and subjected to statistical evaluation. Data was analyzed using the statistical package IBM SPSS Statistics for Windows, Version 26 (IBM Inc., Armonk, NY, USA), and the level of significance was set at p<0.05. Descriptive statistics were performed to assess the mean and standard deviation of the respective groups. The normality of the data was assessed using the Shapiro-Wilk test. Since the data followed normal distribution, parametric tests were used for data analysis. Inferential statistics were used to determine the difference. A one-way ANOVA test followed by the Bonferroni post hoc test was used to check the difference between the pair groups. The Chi-square test was used to detect the difference in proportion.

## Results

The results of the normality tests for four dental bonding materials, 3M Unitek Transbond-XT, 3M ESPE Single Bond Universal, Tetric-N-Bond Universal, and G-Premio Bond (Table 1) indicated that the data for each material adheres to a normal distribution.

**Table 1:** Normality test *P <0.05 is statistically significant

Study groups	Kolmogorov-Smirnov			Shapiro-Wilk		
	Statistic	df	Sig.	Statistic	df	Sig.
3M Unitek Transbond-XT (Group 1)	.224	10	.166^*^	.930	10	.445^*^
3M ESPE Universal Bond Single (Group 2)	.247	10	.126^*^	.858	10	.132^*^
Tetric-N-Bond Universal (Group 3)	.239	10	.109^*^	.864	10	.085^*^
G-Premio Bond (Group 4)	.182	10	.200^*^	.968	10	.873^*^

The study measured the SBS for four bonding agents, revealing that 3M Unitek Transbond-XT had the highest mean SBS at 10.22 MPa, with significant variability (SD = 4.83), while 3M ESPE Single Bond Universal showed the lowest mean at 2.29 MPa (SD = 1.12). Tetric-N-Bond Universal and G-Premio Bond displayed intermediate SBS values of 5.26 MPa (SD = 2.6) and 5.07 MPa (SD = 1.72), indicating moderate variability in their performance (Table 2).

**Table 2:** Descriptive details of shear bond strength values * P<0.05 is statistically significant

Study groups	N	Mean	SD	Std. error	95% confidence interval for mean		Min	Max
					Lower bound	Upper bound		
3M Unitek Transbond-XT	10	10.2194	4.82869	1.52697	6.7651	13.6736	3.28	17.70
3M ESPE Single Bond Universal	10	2.2858	1.12074	.35441	1.4841	3.0876	1.19	4.34
Tetric-N-Bond Universal	10	5.2585	2.65339	.83908	3.3603	7.1566	2.50	10.86
G-Premio Bond	10	5.0692	1.71723	.54304	3.8408	6.2976	2.28	7.82
Total	40	5.7082	4.04397	.63941	4.4149	7.0015	1.19	17.70
F-test				12.61				
P-value				0.0001*				

**Figure 5:**
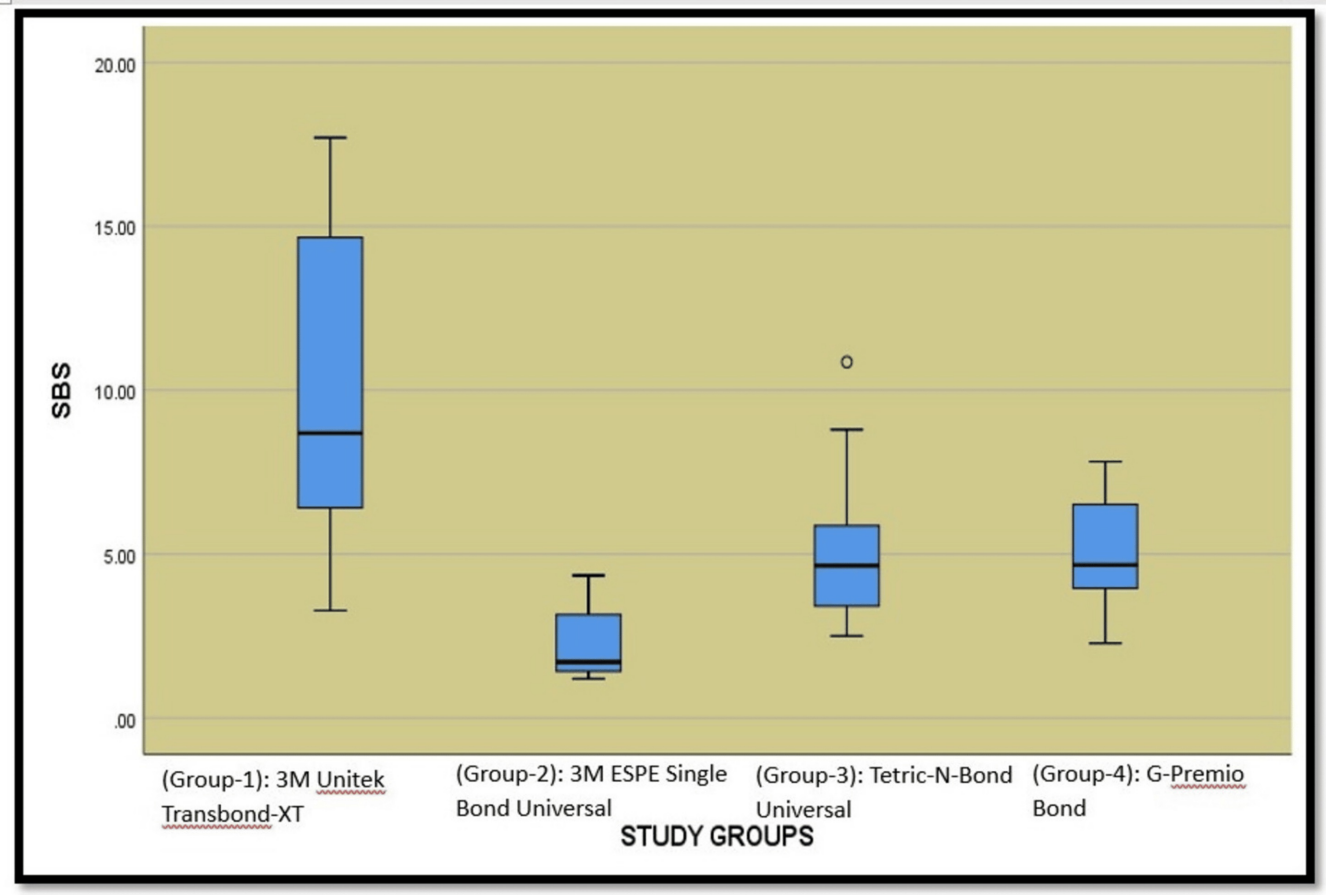
Graphical representation of inter-group comparison of shear bond strength

Figure 5 presents a box plot comparing the SBS of four different bonding agents: 3M Unitek Transbond-XT, 3M ESPE Single Bond Universal, Tetric-N-Bond Universal, and G-Premio Bond. The box plot provides a visual representation of the data distribution, highlighting the median, range, interquartile range (IQR), and potential outliers.

Post-hoc test results (Table 3) showed that 3M Unitek Transbond-XT had statistically significant higher SBS compared to all other adhesives tested. It showed a mean difference of 7.93 with 3M ESPE Single Bond Universal (p = 0.0001), 4.96 with Tetric-N-Bond Universal (p = 0.003), and 5.15 with G-Premio Bond (p = 0.002), all indicating strong statistical significance. In contrast, no significant differences were found between 3M ESPE Single Bond Universal, Tetric-N-Bond Universal, and G-Premio Bond, as the p-values were greater than 0.1, showing these three adhesives performed similarly.

**Table 3:** Post hoc comparison, shear bond strength * P<0.05 is statistically significant

(I) Groups	(J) Groups	Mean difference (I-J)	Std. error	Sig.	95% confidence interval	
					Lower bound	Upper bound
3M Unitek Transbond-XT (Group 1)	3M ESPE Single Bond Universal (Group 2)	7.93356^*^	1.31457	.0001*	4.2633	11.6038
	Tetric-N-Bond Universal (Group 3)	4.96091^*^	1.31457	.003*	1.2907	8.6311
	G-Premio Bond (Group 4)	5.15017^*^	1.31457	.002*	1.4799	8.8204
3M ESPE Single Bond Universal (Group 2)	Tetric-N-Bond Universal (Group 3)	-2.97265	1.31457	.179	-6.6429	.6976
	G-Premio Bond (Group 4)	-2.78339	1.31457	.247	-6.4536	.8868
Tetric-N-Bond Universal (Group 3)	G-Premio Bond (Group 4)	.18926	1.31457	1.000	-3.4810	3.8595

In Table 4, the ARI Index varied significantly among the four bonding agents tested. 3M Unitek Transbond-XT had the highest number of samples with an ARI score of 3, while the eighth-generation adhesives (3M ESPE Single Bond Universal, Tetric-N-Bond Universal, and G-Premio Bond) did not show any samples with this score. Most samples across all groups showed an ARI score of 2. The Chi-square test revealed a value of 19.42 with a p-value of 0.02, indicating a moderately significant difference in ARI scores among the bonding agents used.

**Table 4:** Comparison of ARI between the study groups using Chi-square test ARI: Adhesive Remnant Index * P<0.05 is statistically significant

Scoring		Study groups				Total
		3M Transbond-XT (Group 1)	3M ESPE Single Bond Universal (Group 2)	Tetric-N-Bond Universal (Group 3)	G-Premio Bond (Group 4)	
ARI	0	1	1	2	3	7
		10.0%	10.0%	20.0%	30.0%	17.5%
	1	1	5	4	4	14
		10.0%	50.0%	40.0%	40.0%	35.0%
	2	3	4	4	3	14
		30.0%	40.0%	40.0%	30.0%	35.0%
	3	5	0	0	0	5
		50.0%	0.0%	0.0%	0.0%	12.5%
Total		10	10	10	10	40
		100.0%	100.0%	100.0%	100.0%	100.0%
Chi-square test			19.42			
P-value			0.02*			

**Figure 6:**
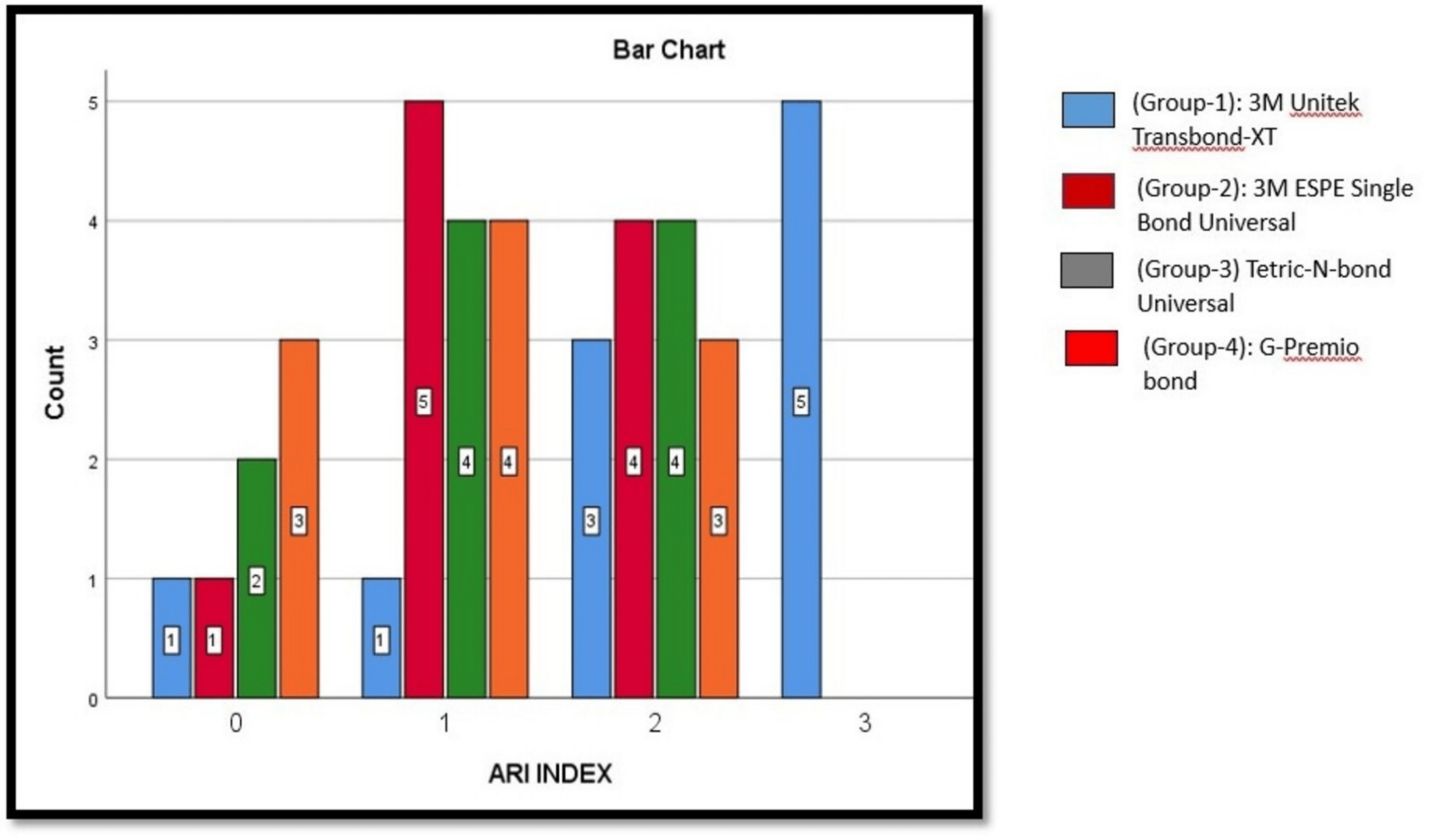
Graphical representation of comparison of ARI between the study groups using Chi-square test ARI: Adhesive Remnant Index

Based on the pictorial representation (Figure 6), the study evaluated different bonding agents by analyzing their ARI values, with lower values (0 and 1) being more desirable. Among the four study groups, 3M Unitek Transbond-XT, 3M ESPE Single Bond Universal, Tetric N-Bond Unitek, and G-Premio Bond, overall, G-Premio Bond and 3M ESPE Single Bond Universal demonstrated better outcomes due to their higher counts at ARI 0 and 1, making them more favorable options for achieving cleaner debonding with minimal adhesive residue. Conversely, 3M Unitek Transbond XT and Tetric-N-Bond Universal appeared to leave more adhesive on the surface, as indicated by their higher counts at ARI 2 and 3, making them less ideal in this context.

## Discussion

The bonding of orthodontic brackets to tooth enamel is a crucial step in fixed orthodontic therapy, as it significantly impacts treatment efficiency, patient comfort, and oral hygiene. The development of bonding techniques has evolved since the introduction of the acid-etch technique by Buonocore, which was adapted by Bowen, Tavas and Watts, and later adopted by Newman et al. for orthodontic bracket bonding [17-20]. This approach eliminated the need for traditional metal bands, thus reducing treatment time, improving patient comfort, and increasing the aesthetic appeal of orthodontic treatment. The introduction of direct bonding also reduced gingival irritation, made plaque removal easier, and allowed for earlier detection of dental caries. These advantages have improved the overall success of orthodontic treatment [21].

For orthodontic therapy to be effective, the bond strength between the bracket and the tooth enamel must be sufficient to withstand masticatory forces and mechanical stresses exerted by the archwire. The adhesive bond must resist forces during treatment without causing bracket failure, as repeated re-bonding not only delays treatment but can also damage tooth enamel. Several factors influence the efficacy of bracket bonding, including the type of adhesive used, the enamel conditioning process, the nature of the bracket surface, and the operator's technique. As such, optimizing bond strength remains a key objective in orthodontic treatment [22-25].

In this study, the SBS of four different adhesives was compared: 3M Unitek Transbond-XT (fifth generation), 3M ESPE Single Bond Universal (eighth generation), Tetric-N-Bond Universal (eighth generation), and G-Premio Bond (eighth generation). The study aimed to assess the effectiveness of eighth-generation adhesives in bonding orthodontic brackets, and one-way analysis of variance (ANOVA) was performed to compare the SBS of four different dental bonding materials: 3M Unitek Transbond-XT, 3M ESPE Single Bond Universal, Tetric-N-Bond Universal, and G-Premio Bond. The 3M Unitek Transbond-XT showed the highest SBS values of 10.2±4.8 MPa among all four bonding agents used in the current study. Bilal compared the SBS values for conventional-etched and self-etched adhesives [1]. He reported the overall mean SBS for the conventional etched group (14.71 MPa,) which was significantly greater than the self-etched adhesive group (9.24 MPa) (P = 0.024). Lower SBS values for 3M Unitek Transbond-XT (9.38 ± 6.02 MPa) were reported by Bhattacharjee et al. [26]. Almokaddam et al. also determined the SBS values of the 3M Unitek Transbond-XT bonding agent (12.28 MPa), around the same values reported in previous studies [15].

Our current study showed the mean SBS values for the eight-generation adhesives as 2.2±1.12 MPa for 3M ESPE Single Bond Universal, 5.2±2.6 MPa for Tetric-N-Bond Universal, and 5.06±1.71 MPa for G-Premio Bond. A similar study comparing eighth-generation with fifth-generation was done by Khalid et al., where they compared 3M Unitek Transbond-XT, a fifth-generation adhesive, with Futurabond DC (Voco GmbH, Cuxhaven, Germany), an eighth-generation adhesive [8].But, here the universal adhesive showed greater SBS values of 34.9±4.02 MPa than the conventional 3M Unitek Transbond-XT (16.10 MPa±3.34MPa), which might be attributed to different mechanical and chemical properties of a different eight-generation bonding agent Futurabond DC used in this study.

The 3M ESPE Single Bond Universal bonding agent used in our current study in the second group gave the least SBS values of 2.2MPa±1.12 MPa, while the G-Premio Bond bonding agent showed the SBS values of 5.06±1.71 MPa. These two bonding agents, when used after etching, gave better results in a study done by Hoseinifar et al. [27]. The premolars were etched with 37% phosphoric acid before bonding. Here, the first group of premolars was bonded using 3M Unitek Transbond-XT, which was applied only to the enamel in the conventional group. In the second group, 3M Unitek Transbond-XT was applied to both the enamel and the bracket base, 3M ESPE Single Bond Universal bonding agent to the third group, and G-Premio Bond to the last group. The SBS values for the 3M ESPE Single Bond Universal bonding agent (25.4±8.MPa) were much higher than those in our present study, which might be mainly due to the lack of etching in our study.

Previous studies by Sfondrini et al. have reported the usage of 3M ESPE Single Bond Universal bonding agent/3M ESPE Scotchbond Universal in total-etch mode in bonding the lingual retainers in comparison with 3M Unitek Transbond-XT primer [28]. The SBS values of 3M ESPE Scotchbond Universal obtained were similar to the conventional 3M Unitek Transbond-XT in their study, but higher than the readings obtained in our current study, which were 2.2 MPa±1.12 MPa. These higher values may again be attributed to the lack of etching done in our current study.

The third group in our present study was bonded using Tetric-n-Bond Universal, resulting in SBS values of 5.25±2.6 MPa. Almokaddam et al. have compared the SBS values of 3M Unitek Transbond-XT (USA) in the first group, Beauty Ortho bond (Shofu Inc., Kyoto, Japan) in the second group, and Tetric-N-Bond universal bonding agent (Vivapen) (USA) with and without etching with 37% phosphoric acid in the third and fourth groups, respectively [15]. Tetric-N-Bond universal with etching showed 12.66 MPa of SBS, and without etching showed a decrease in their SBS value, corresponding to our current study.

Usage of Universal adhesives in self-etch mode has been demonstrated in the past by Sharma et al. [9] in their studies where they compared the SBS of Transbond Plus (3M Unitek, Monrovia, CA, USA) and Xeno-V (Dentsply, Konstanz, Germany) self-etching primer with Rely-a-Bond (self-cure adhesive, Reliance Orthodontic Product, Inc., Illinois, USA) primer and Transbond-XT primer (light-cure adhesive, 3M Unitek, Monrovia, CA, USA) using conventional etching which also showed statistically significant differences in the values among all the bonding agents with highest values for the conventional 3M Unitek Transbond-XT adhesive than the self-etching primers corresponding to the present study The values cannot be directly compared with the present study as the bonding primers and adhesives used were different in composition with those used in our study.

A similar difference was seen in a study done by Bhattacharjee et al., where they used 3M Unitek Transbond-XT in the conventional group with etching performed before bonding and Transbond Plus in the second group in self-etch mode [26]. Here, the self-etch adhesive showed less SBS values of 6.91 ± 3.58 MPa than for the conventional group (9.38 ± 6.02) MPa.

Previously, Beltrami et al. compared the SBS values for six universal adhesives: Futurabond M+ (VOCO GmbH, Cuxhaven, Germany), Scotchbond Universal DCA (3M ESPE, St. Paul, Minnesota, USA), Adhese Universal (Ivoclar Vivadent, Schaan, Liechtenstein), Clearfil Universal Bond (Kuraray Noritake Dental Inc, USA), GBU-500 (GC Corporation, Tokyo, Japan), Peak Universal Bond (Ultradent, Salt Lake City, Utah, USA), and two self-etch adhesives, Optibond XTR (Kerr, Brea, California, USA) and Clearfil SE Bond 2 (Kuraray, Tokyo, Japan) [29]. All eight agents were used to bond the brackets to two groups of incisors. The first group was etched with 37% orthophosphoric acid, and in the second group, these bonding agents were used in self-etch mode [29]. All the adhesives showed lower values in the second group, which was not pretreated using 37% phosphoric acid, which was quite similar to the results seen in the present study. In our study, too, the results for the group treated with 37% phosphoric acid (3M Unitek Transbond-XT) showed higher values with 10.42±4.8 MPa as compared to the adhesives: Tetric-N-Bond Universal, 3M ESPE Single Bond Universal, and G-Premio Bond, which showed 5.2±2.6 MPa, 2.2±1.2 MPa, and 5.06±1.7 MPa, respectively.

A post hoc analysis of the SBS values showed that 3M Unitek Transbond-XT significantly outperformed all three eighth-generation adhesives (p < 0.005). However, no significant difference was observed between Tetric-N-Bond Universal and G-Premio Bond (p > 0.05), indicating that these two adhesives provided similar bond strength and could be considered interchangeable in clinical practice. Although the eighth-generation adhesives displayed lower SBS values, they still fell within the clinically acceptable range and demonstrated consistent performance across samples.

In addition to SBS, the study also evaluated the failure mode of the bonding agents using the ARI, which allows for the assessment of the amount of adhesive remaining on the tooth surface after debonding. The ARI provides insights into the nature of bond failure, whether it occurs at the tooth-adhesive interface or at the bracket-adhesive interface. The results showed that 3M Unitek Transbond-XT had the highest percentage of ARI score 3 (50%), indicating that the failure occurred primarily at the bracket-adhesive interface, leaving adhesive on the tooth. In contrast, the self-etching adhesives, such as 3M ESPE Single Bond Universal, Tetric-N-Bond Universal, and G-Premio Bond, displayed higher proportions of ARI scores 1 and 2, which suggests that failure occurred within the adhesive layer itself. This type of failure is preferable in clinical settings because it makes cleaning the tooth surface easier, as less adhesive remains on the enamel.

These findings align with previous studies, such as Yadala et al., which reported that self-etching adhesives tend to result in more favorable ARI scores, indicating less adhesive residue after debonding [21]. While the SBS of self-etching adhesives was lower than that of conventional adhesives, the ARI scores suggest that self-etch adhesives might offer advantages in terms of easier cleanup and reduced enamel damage. This trade-off between bond strength and ease of cleanup makes eighth-generation adhesives, despite their lower SBS, a clinically viable alternative, especially in cases where bond failure is unlikely or easily managed.

Although 3M Unitek Transbond-XT provided the highest SBS in this study, the lower variability in the results of the eighth-generation adhesives may also be seen as an advantage, particularly in clinical settings where predictability is important. The relatively consistent performance of Tetric-N-Bond Universal and G-Premio Bond, despite their lower bond strengths, indicates that these adhesives may be more reliable in terms of clinical outcomes.

This study is not without limitations. In vitro studies, while useful, do not fully replicate the complexities of the oral environment, where factors like masticatory forces, saliva, and temperature fluctuations affect adhesive performance. Further, in vivo studies are needed to validate these results and explore the long-term clinical effectiveness of eighth-generation adhesives. Additionally, the use of different bonding agents in real-world conditions, including variations in technique and operator experience, may influence the bond strength and failure modes observed.

This study demonstrates that while 3M Unitek Transbond-XT continues to offer the highest SBS, eighth-generation adhesives like Tetric-N-Bond Universal and G-Premio Bond are promising alternatives. They provide acceptable bond strengths and, importantly, demonstrate favorable ARI scores, which may contribute to easier cleanup and reduced enamel damage during debonding. These adhesives, despite their lower SBS, could become valuable tools in orthodontic practice, particularly for clinicians prioritizing efficiency and patient comfort. Further clinical studies will help to confirm the applicability and long-term performance of these materials in real-world orthodontic settings.

## Conclusions

In conclusion, this study evaluated the shear bond strength (SBS) and Adhesive Remnant Index (ARI) of different adhesives used for bonding metal orthodontic brackets to enamel. The 3M Unitek Transbond-XT showed the highest SBS (10.2 ± 4.8 MPa), significantly outperforming other adhesives. The 3M ESPE Single Bond Universal had the lowest SBS (2.2 ± 1.12 MPa), below the recommended bond strength for effective orthodontic bonding. Tetric-N-Bond Universal and G-Premio Bond, with SBS values of 5.2 ± 2.6 MPa and 5.06 ± 1.07 MPa, showed similar performance. The 3M Unitek Transbond-XT had significantly higher SBS than the other adhesives, while Tetric-N-Bond Universal and G-Premio Bond exhibited less adhesive residue (lower ARI scores), suggesting easier cleanup and less enamel damage. Despite simplifying the bonding procedure, the eighth-generation adhesives (Tetric-N-Bond Universal and G-Premio Bond) showed lower bond strength than 3M Unitek Transbond-XT, limiting their effectiveness for orthodontic bonding. Thus, while these newer adhesives offer benefits in enamel protection and cleanup, their lower SBS may not meet the required standards for strong and durable orthodontic bonding.
